# Occipitocervical Hemolymphangioma in an Adult with Neck Pain and Stiffness: Case Report and Literature Review

**DOI:** 10.1155/2017/7317289

**Published:** 2017-12-07

**Authors:** Yongchao Li, Xing Zhang, Xiaodong Pang, Liang Yang, Baogan Peng

**Affiliations:** ^1^Department of Orthopedics, Gaoyou People's Hospital Affiliated to Soochow University, Yangzhou, Jiangsu 225600, China; ^2^Department of Spinal Surgery, General Hospital of Armed Police Force, Beijing 100039, China

## Abstract

**Introduction:**

Hemolymphangioma is an extremely rare malformation of the lymphatic and blood vessels. A limited number of hemolymphangioma cases occurring in the pancreas, extremities, spleen, and other organs have been reported until September 2017. To the best of our knowledge, no cases of hemolymphangioma in the occipitocervical region have been reported in the literature.

**Case Presentation:**

We reported the case of a 23-year-old male patient with an occipitocervical lesion presenting atypically as neck pain and stiffness over a period of five months. Although hemolymphangioma has historically demonstrated a female predilection (2.25 : 1 female to male) and presentation in the third to fourth decades of life, this case is an atypical manifestation occurring in a young male patient. The clinical characteristics and management choices of this uncommon case of hemolymphangioma in the occipitocervical region are discussed, and a review based on the available literature is also presented.

**Conclusion:**

Hemolymphangioma of the occipitocervical region is an uncommon presentation of a rare lesion. Although rare, hemolymphangioma should be considered a differential diagnosis for masses occurring in the occipitocervical region. Complete surgical resection is the treatment of choice and affords a good prognosis.

## 1. Introduction

Hemolymphangioma is a malformation of both the lymphatic and vascular systems. Clinically, the onset of hemolymphangioma can vary from a slowly growing cyst over a period of years to an aggressive enlarging tumor, without invasive ability [1]. A limited number of hemolymphangioma cases occurring in the pancreas, extremities, spleen, and other organs have been reported until September 2017 [1–25]. To the best of our knowledge, the present study describes the first case of hemolymphangioma in the occipitocervical region, which appears to be a rare site of presentation of a rare neoplasm and reviews the clinical characteristics and management choices of this condition based on the existing available literature.

## 2. Case Presentation

A 23-year-old man presented with a posterior occipitocervical subcutaneous mass that had been present since birth and had been growing for years. He complained of neck pain and stiffness for approximately 5 months, with no symptoms of extremity numbness or pain. There was no history of trauma or weight loss and was no family history of cancer. Physical examination revealed a partially spherical lesion, compressible, with mild tenderness. Laboratory data showed no infectious or inflammatory findings.

**Figure 1:**
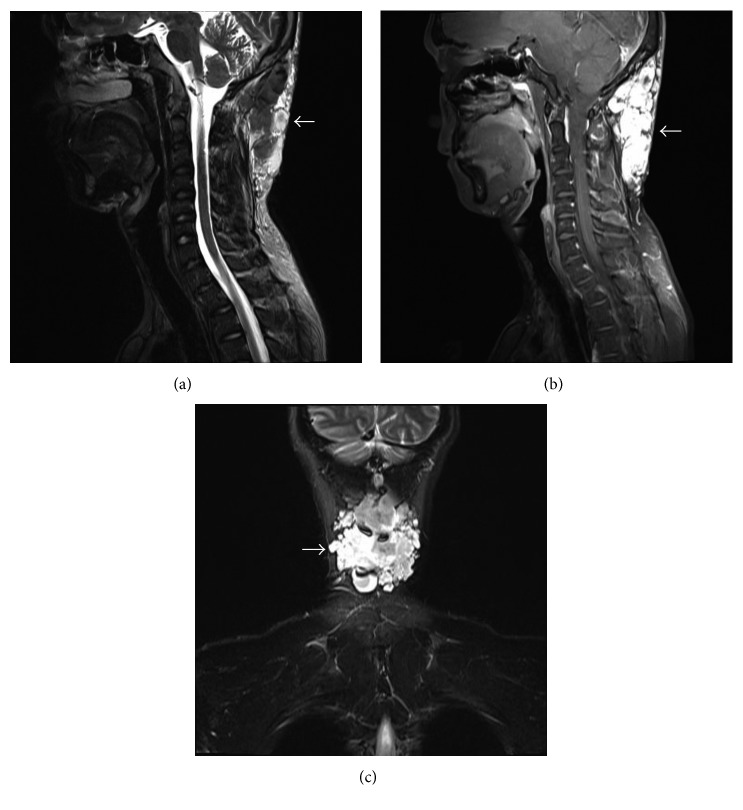
MRI demonstrated a mass (arrows) in the occipitocervical subcutaneous tissue with uneven signal intensity on (Figure 1(a)) sagittal T2-weighted, (Figure 1(b)) T2-weighted fat suppressed, and (Figure 1(c)) coronal enhanced images.

Magnetic resonance imaging (MRI) examination was performed preoperatively, in order to establish the extent of the tumor and define its association with the surrounding tissues. The occipitocervical MRI scan (Figure 1) showed an approximate lesion measuring 11.2 × 3.5 × 6.9 cm with an irregular shape, unclear boundary, and uneven signal intensity on sagittal T2-weighted imaging (WI), dominantly high signal intensity on T2-WI fat suppressed, and markedly heterogeneous enhancement on coronal enhanced scan in the occipitocervical subcutaneous tissue.

**Figure 2:**
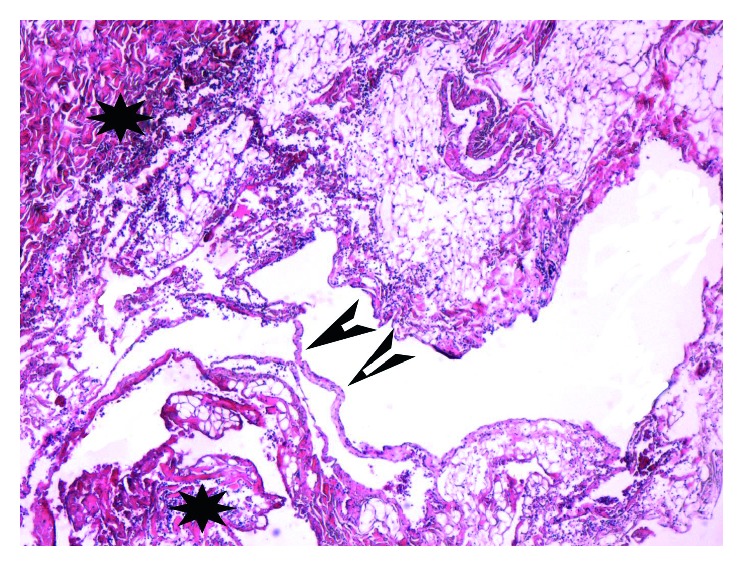
Histological analysis of specimen (hematoxylin and eosin stain; magnification ×100) showed abnormal lymphatic (black arrowheads) and blood vessels (black stars) with polycystic spaces.

The patient was admitted for surgery. During the operation, the boundary of the mass was unclear. Macroscopically, the mass spaces between all mass measurements were 10.8 × 3.4 × 6.2 cm. It was polycystic and soft in consistency. Histopathological examination (Figure 2) revealed abnormal lymphatic and blood vessels with polycystic spaces, thin wall, and dyed red lymph and blood cells within the lumen, which confirmed a diagnosis of hemolymphangioma. The postoperative course of the patient was uneventful, and the patient was discharged 2 weeks following the surgery. At 8 months of follow-up, he had no visible recurrence of the subcutaneous lesion and no evidence of neck pain and stiffness.

## 3. Literature Review

In preparation of this case report, a review of the existing medical literature was performed with the PubMed database, using the following keyword: “Hemolymphangioma” to identify all possible studies published up to September 2017. References from these articles were also reviewed. After carefully reviewing and summarizing each published article, we selected 25 original studies with 26 case reports [1–25]. We find this tumor typically appearing in adult patients ranging in age from 2 months to 62 years, with an average age of 31.4 years. Nearly 61.5% (16/26) of all cases occurred in patients over the age of 20. Hemolymphangioma is most common in female patients, with the sex distribution of male to female 1 : 2.25. The clinical manifestation is not typical, whereby it can be hidden for a long time. The reported anatomic location of hemolymphangioma has been 23.1% (6/26) in the pancreas, 15.3% (4/26) in the extremities, 11.5% (3/26) in the spleen, and almost 69.2% (18/26) in internal or visceral locations. To date, no reports documenting recurrence have been reported after short-term follow-up. The clinical characteristics and management choices of all 26 patients with hemolymphangioma are shown in Table 1.

## 4. Discussion

Hemolymphangioma is thought to originate from the mesenchymal tissue [15] and typically found by palpation or with symptomatic compression of nearby anatomic structures [21]. It may be divided into primary and secondary lymphatic vascular tumors. The primary tumor is considered to be a congenital malformation of the lymphatic vascular system, possibly formed due to obstruction of the venolymphatic communication between dysembryoplastic vascular tissue and the systemic circulation. By contrast, the secondary tumor is caused by poor lymph drainage and lymphatic damage resulting from surgery or trauma [15, 20].

This tumor is a benign hamartoma of blood and lymphatic vessels with a predilection for the pancreas, spleen, and lower extremity and, less commonly, in occipitocervical lesion. In general, hemolymphangiomas are large masses of varying sized cystic cavities with thin walls, having multiple thin septa and containing fluid similar to hemorrhagic fluid, and rarely of clear lymphatic nature. The tumor size varies due to the anatomical location and relationship to the neighboring tissues [15]. The majority of small tumors remain asymptomatic for a long period of time. As the tumor develops, discomfort occurs, which is mainly caused by surrounding or infiltrating the neighboring tissues or other major structures [15]. In the current case, the main complaints were neck pain and stiffness for approximately five months, without symptoms of extremity numbness or pain. In clinical examinations, they are usually described as soft and compressible masses, loculated in pattern [20]. The histopathological examination suggests that the tumors often consist of abnormal lymphatic and blood vessels with polycystic spaces, and the thin-walled cystic lesion has connective septa covered by endothelium [7]. However, all cases in the literature had no abnormal laboratory findings as did our patient.

The impossibility to preoperatively define the histological type of the tumor explains the difficulties to reach a correct differential diagnosis. Biopsy should not be performed because of the high risk of massive bleeding. It is very important and crucial for radiologists to recognize these lesions and establish an accurate diagnosis so as to avoid a biopsy, which could cause severe hemorrhage [6]. In the present study, an occipitocervical MRI showed a lesion with irregular shape, unclear boundary, and uneven signal intensity on sagittal T2-WI, dominantly high signal intensity on T2-WI fat suppressed, and markedly heterogeneous enhancement on coronal enhanced scan in the occipitocervical subcutaneous tissue. These observations may indicate the presence of a lower number of tortuous blood vessels and water-based substance in the lesion, which was then confirmed during surgery. Imaging examinations, including ultrasound, computed tomography, and MRI scans, are useful in order to confirm the diagnosis, identify the tumor nature, and observe its extension and association with the surrounding tissues, assisting the selection of the surgical strategy [20]. However, an accurate diagnosis of the tumor cannot be preoperatively established in spite of modern imaging techniques and can be postoperatively based on histological evidence.

Most researchers believe that hemolymphangioma is commonly a benign disease and has no invasive ability [1, 2, 5–11, 13–23]. But some studies recently found that it can invade the adjacent structures [3, 4, 12]. Toyoki et al. discovered that the tumor from pancreas invaded to the duodenum to cause the duodenal bleeding [3]. In 2009, Sun and colleagues reported that the giant tumor originated in pancreas, infiltrated the transverse mesocolon and greater omentum, and tightly adhered to duodenum and superior mesenteric artery [4]. Recently, Zhang et al. found multiple hemolymphangioma of the visceral organs and reported the retroperitoneal tumor extending to the left colon and small bowel mesentery [12].

The optimal treatment strategy for this lesion remains controversial. Surgical resection appears to be the most effective treatment for hemolymphangioma, especially when the tumor increases in size and creates pressure on the surrounding tissues. To prevent recurrence, during surgery, a thorough radical resection may be necessary. Furthermore, with tumor adhesion or invasion to the surrounding organs, the removal of adjacent organs needs to be considered [3]. Tumor removal may also be associated with complications such as infection, fistula, and hemorrhage [4, 16]. Beninson et al. successfully treated hemolymphangioma in a neonate using a pressure dressing initially followed by combined compression therapy [9]. Wang and colleagues reported a case of successful treatment of hemolymphangioma of the tongue with a variable-pulse 595 nm pulsed-dye laser [23]. All cases in the existing literature had good clinical outcomes as did our case. The risk of recurrence seems very low, and no documented reports of recurrence have been reported in the literature. In addition, so far, no case of malignant transformation was reported. However, careful follow-up with MRI or ultrasound is recommended.

## 5. Conclusion

Hemolymphangioma of the occipitocervical region is an uncommon presentation of a rare lesion. Although rare, hemolymphangioma should be considered a differential diagnosis for masses occurring in the occipitocervical region. Complete surgical resection is the treatment of choice and affords a good prognosis.

## Figures and Tables

**Table 1:** The clinical characteristics and management choices of 26 patients with hemolymphangioma [1–25].

Case	Publication year	Age (y)/sex	Localization	Size (cm)	Chief complaint	Treatment	Follow-up (months)	Recurrence	Evolution
1 [8]	1979	20/F	Lower extremity	NA	Painful swelling, chest pain, and pulmonary embolism	Urokinase for a short period; prolonged maintenance therapy was with a low dose of heparin	NA	NA	Favourable
2 [9]	1988	1.5/F	Lower extremity	4.0 × 5.0	Not reported	Pressure dressing and combined compression therapy	9	Not reported	Favourable
3 [13]	1993	NA/M	Abdomen	13.0	Not reported	Autopsy	NA	NA	Stillborn
4 [14]	1996	2/F	Esophagus	9.5 × 2.5 × 2.0	Acute dyspnea	Endoscopically	7	Not reported	Favourable
5 [2]	2003	53/F	Pancreas	4.0 × 3.0	Abdominal pain and weight loss	Pancreatoduodenectomy	NA	NA	Favourable
6 [23]	2005	15/F	Tongue	NA	Pain and bleeding	Pulsed-dye laser	10	Not reported	Favourable
7 [3]	2008	53/M	Pancreas	NA	Severe anemia due to gastrointestinal bleeding	Pylorous-preserving pancreatoduodenectomy	NA	Not reported	Favourable
8 [4]	2009	20/F	Pancreas	18.0 × 16.0 × 12.5	Epigastric discomfort	Pancreatoduodenectomy	26	Not reported	Favourable
9 [1]	2010	0.16/F	Lower extremity	4.5	Asymptomatic	Surgical excision	12	Not reported	Favourable
10 [1]	2010	5/M	Lower extremity	2.1 × 1.8 × 0.5	Asymptomatic	Surgical excision	12	Not reported	Favourable
11 [15]	2012	57/F	Chest wall	9.0 × 9.0 × 5.0	Chest tightness, shortness of breath, cough, and expectoration	Thoracotomy	3	Not reported	Favourable
12 [16]	2012	57/F	Small intestine	5.0 × 4.0	Recurrent melena	Partial resection of the small intestine	12	Not reported	Favourable
13 [17]	2013	53/F	Stomach	1.5	Bloody vomiting and epigastric pain	Endoscopic therapy	NA	NA	Favourable
14 [18]	2013	37/M	Rectum	20.0 × 8.0 × 8.0	Rectal bleeding and tenesmus	Low anterior resection of the rectosigmoid colon with handsewn transanal coloanal anastomosis	12	Not reported	Favourable
15 [5]	2013	39/F	Pancreas	10.0 × 7.0	Abdominal pain	Pancreatic body-tail resection combined with splenectomy	NA	Not reported	Favourable
16 [19]	2014	24/F	Duodenum	4.0 × 1.5	Severe anemia	Surgical excision	NA	Not reported	Favourable
17 [6]	2014	52/F	Pancreas	8.0 × 6.5 × 6.0	Abdominal pain and epigastric discomfort	Pylorus preserving pancreatoduodenectomy	NA	NA	Died
18 [10]	2014	12/M	Spleen	15.7 × 8.5	Abdominal pain	Laparoscopic partial splenectomy	12	Not reported	Favourable
19 [7]	2015	57/F	Pancreas	8.0 × 6.0 × 4.5	Epigastric discomfort	Surgical excision	2	Not reported	Favourable
20 [11]	2015	62/F	Spleen	11.0 × 6.0 × 3.0	Abdominal pain	Total splenectomy	12	Not reported	Favourable
21 [20]	2015	17/M	Waist	12.0 × 6.0 × 6.0	Back pain	Surgical excision	7	Not reported	Favourable
22 [12]	2015	25/F	Spleen, retroperitoneum	28.0 × 24.0 × 15.0	Progressive splenomegaly	Splenectomy	NA	NA	Favourable
23 [21]	2015	15/F	Paraspinous	NA	Scoliosis	Posterior spinal fusion	12	Not reported	Favourable
24 [22]	2016	48/F	Posterior mediastinum	3.1 × 2.4	Shortness of breath and chest tightness	Thoracic surgery	12	Not reported	Favourable
25 [24]	2016	3/M	Greater omentum	20 × 15 × 6	Abdominal pain	Surgical excision	6	Not reported	Favourable
26 [25]	2017	57/M	Rectum, sigmoid	25	Rectorrhagia	Surgical excision	6	Not reported	Favourable

NA, the data were not available.
